# Sensor‐based gait analysis in atypical parkinsonian disorders

**DOI:** 10.1002/brb3.977

**Published:** 2018-05-07

**Authors:** Cecilia Raccagni, Heiko Gaßner, Sabine Eschlboeck, Sylvia Boesch, Florian Krismer, Klaus Seppi, Werner Poewe, Bjoern M. Eskofier, Juergen Winkler, Gregor Wenning, Jochen Klucken

**Affiliations:** ^1^ Department of Neurology Medical University of Innsbruck Innsbruck Austria; ^2^ Department of Molecular Neurology University Hospital Erlangen Friedrich‐Alexander University Erlangen‐Nürnberg (FAU) Erlangen Germany; ^3^ Machine Learning and Data Analytics Lab Friedrich‐Alexander University Erlangen‐Nürnberg (FAU) Erlangen Germany

**Keywords:** atypical parkinsonian disorders, multiple system atrophy, parkinson's disease, progressive supranuclear palsy, sensor‐based gait analysis

## Abstract

**Background and Objectives:**

Gait impairment and reduced mobility are typical features of idiopathic Parkinson's disease (iPD) and atypical parkinsonian disorders (APD). Quantitative gait assessment may have value in the diagnostic workup of parkinsonian patients and as endpoint in clinical trials. The study aimed to identify quantitative gait parameter differences in iPD and APD patients using sensor‐based gait analysis and to correlate gait parameters with clinical rating scales.

**Subjects and Methods:**

Patients with iPD and APD including Parkinson variant multiple system atrophy and progressive supranuclear palsy matched for age, gender, and Hoehn and Yahr (≤3) were recruited at two Movement Disorder Units and assessed using standardized clinical rating scales (MDS‐UPDRS‐3, UMSARS, PSP‐RS). Gait analysis consisted of inertial sensor units laterally attached to shoes, generating as objective targets spatiotemporal gait parameters from 4 × 10 m walk tests.

**Results:**

Objective sensor‐based gait analysis showed that gait speed and stride length were markedly reduced in APD compared to iPD patients. Moreover, clinical ratings significantly correlated with gait speed and stride length in APD patients.

**Conclusion:**

Our findings suggest that patients with APD had more severely impaired gait parameters than iPD patients despite similar disease severity. Instrumented gait analysis provides complementary rater independent, quantitative parameters that can be exploited for clinical trials and care.

## 1 INTRODUCTION

Gait impairment is a major motor symptom of idiopathic Parkinson's disease (iPD). It is even more prominent in patients with atypical parkinsonian disorders (APDs) including multiple system atrophy (MSA) and progressive supranuclear palsy (PSP), leading to an impaired quality of life and shorter latency from symptom onset to recurrent falls (Wenning et al., 1999). The clinical severity including motor and non‐motor symptoms of parkinsonian disorders is commonly defined by semi‐quantitative rating scales such as the Movement Disorder Society Unified Parkinson's Disease Rating Scale (MDS‐UPDRS) (Goetz, 2010), Unified MSA Rating Scale (UMSARS) (Wenning et al., 2004), and PSP Rating Scale (PSP‐RS) (Golbe & Ohman‐Strickland, 2007), showing good to excellent construct and face validity. However, non‐metric rating scales do not provide objective, metric measures for clinical studies. These considerations led to the development of complementary quantitative assessment tools of motor (in particular locomotor) function in iPD (Espay et al., 2016; Maetzler, Klucken, & Horne, 2016; Lees, Hardy, & Revesz, 2009).

From a biomechanical point of view, gait is a highly regular and cyclic movement, which makes it ideal for automated sensor‐based detection and subsequent quantitative and qualitative analysis with a high biomechanical resolution. Body‐worn inertial measurement units, comprising of the biosensors 3D accelerometer, gyroscope, and magnetometer are able to objectively measure changes of gait patterns in PD (Barth et al., 2015; Klucken et al., 2013; Kegelmeyer, Parthasarathy, Kostyk, White, & Kloos, 2013; Schlachetzki et al., 2017). A new era in medical engineering is emerging, where objective real‐time motion metrics in iPD could be obtained in virtually any environmental scenario by placing lightweight wearable sensors in the patient's clothes, and connecting them to a medical database through mobile devices such as cell phones or tablets (Pasluosta, Gassner, Winkler, Klucken, & Eskofier, 2015; Schlachetzki et al., 2017). This approach provides comprehensive, objective and metric data, enabling an assessment for clinical studies in iPD which is free from the confounds of observer bias (Espay et al., 2016; Lees et al., 2009). It also allows continuous patient monitoring even in unsupervised, habitual environments (Del Din, Godfrey, Mazzà, Lord, & Rochester, 2016). Furthermore, bringing healthcare technology into clinical practice might improve diagnostic accuracy and provide the base for multidisciplinary care concepts using rater‐independent quantitative measures of motor signs.

Whereas numerous studies showed the feasibility of wearable sensors in iPD, there are only a few studies about the use of instrumented technology in APD (Baston, Mancini, Schoneburg, Horak, & Rocchi, 2014; Hatanaka et al., 2016; Sale et al., 2014; Sanchez‐Ferro et al., 2016). To our knowledge, there are no published studies that compare results of instrumented gait analysis between cohorts of MSA, PSP, iPD patients, and healthy subjects.

The goal of the present cross‐sectional study was to test whether a mobile, objective, and simple to use gait assessment system is able to detect differences in gait parameters between APD (Parkinson‐variant MSA = MSA‐P, PSP) vs. iPDs patients vs. controls. Also, to decipher the clinical value of the gait parameter alterations, we correlated them to clinical ratings in standardized scales (MDS‐UPDRS part 3 = MDS‐UPDRS‐3 (Goetz, 2010; Movement disorder society task force, 2003), UMSARS part 1 and 2 (=UMSARS 1/2) (Wenning et al., 2004), and PSP‐RS (Golbe & Ohman‐Strickland, 2007). We not only identified distinct gait parameters that differed between matched patient/control cohorts, but also their correlation to the different clinical characteristics, underlining the complementary diagnostic value of sensor‐based gait assessment.

## 2 SUBJECTS AND METHODS

In all, 50 patients were enrolled in the outpatient clinics of the Department of Neurology at the Medical University Hospital Innsbruck, Austria, and the Department of Molecular Neurology at the University Hospital Erlangen, Germany. iPD and APD (probable MSA‐P *n* = 11, possible MSA‐P *n* = 2, probable PSP *n* = 12 patients) were defined according to standard diagnostic criteria (Movement disorder society task force, 2003; Gilman et al., 2008; Litvan et al., 1996). Exclusion criteria consisted of non‐PD‐related gait impairments (e.g., spinal or orthopedic surgery), spasticity, stroke, neuropathy, myelopathy, hydrocephalus, and severe dementia. All patients were investigated in stable ON medication without the presence of motor fluctuations. This study has been approved by the local ethics committees in Erlangen, Germany and Innsbruck, Austria (IRB‐approval‐Re. No. 4208, 21.04.2010, IRB, Medical Faculty, Friedrich‐Alexander‐Universität Erlangen‐Nürnberg, Germany, IRB‐approval‐Re. No 0365, 27.04.2015, Medical Faculty Innsbruck, Austria) and have therefore been performed in accordance with the ethical standards laid down in the 1964 Declaration of Helsinki and its later amendments.

iPD, MSA‐P, and PSP patients were rated by MDS‐UPDRS‐3. Some iPD patients were rated by UPDRS‐3 (Rampp et al., 2015). For these patients, the conversion method of UPDRS‐3 into MDS‐UPDRS‐3 was applied (Hentz et al., 2015). Furthermore, MSA‐P patients were scored using UMSARS (Wenning et al., 2004) and PSP patients were assessed by PSP‐RS (Golbe & Ohman‐Strickland, 2007).

Gait was evaluated in 25 APD and 25 pairwise matched iPD patients matched by age, gender, age at onset, Hoehn and Yahr stage, and Levodopa equivalent daily dose (LEDD) (Tomlinson et al., 2010) as well as in 25 matched healthy individuals (matched by age and gender) using sensor‐based gait analysis. The 25 iPD patients were selected from a larger stratified patient cohort (*n* = 406) visiting the Movement Disorders outpatient clinic of the Department of Molecular Neurology at the University Hospital Erlangen, Germany between July 2014 and March 2016 (Schlachetzki et al., 2017). Walking performance was captured using a sensor‐based gait analysis system. This system consists of wearable SHIMMER 2 sensors (Shimmer Research Ltd., Dublin, Ireland) laterally attached to the posterior lateral portion of both shoes. Gait signals were recorded within a (tri‐axial) accelerometer range of ±6 g (sensitivity 300 mV/g), a gyroscope range of ±500 degree/s (sensitivity 2 mV/degree/s), and a sampling rate of 102,4 Hz. Sensor signals were transmitted via Bluetooth^®^ to a tablet computer and stored for subsequent data analysis (Kegelmeyer et al., 2013). Inertial sensor data were processed with a pattern recognition algorithm for calculating clinically relevant spatiotemporal gait parameters (e.g., stride length, gait speed, maximum toe clearance) (Barth et al., 2015; Rampp et al., 2015). Participants performed standardized overground walking tests on a 10‐m long corridor in the hospital in self‐chosen walking speed. Only straight strides were automatically detected by the stride detection algorithm (Barth et al., 2015) and used for gait parameter calculations as described (Rampp et al., 2015). Calculated gait speed, stride length, cadence, and maximum toe clearance were normalized to the height of the participants.

A one‐way ANOVA was used to detect differences in spatiotemporal gait parameters between groups. For all parameters that were compared between the three groups (iPD, APD, Controls), Bonferroni post‐hoc test was used to account for multiple comparisons. Mann‐Whitney‐U Test was performed for the subgroup analysis in MSA patients in which participants with and without impairment in body sway and walking were compared in terms of gait parameters. In a second step, correlation analysis was used to evaluate associations between clinical scores (MDS‐UPDRS, UMSARS, and PSP‐RS) and spatiotemporal gait parameters. Spearman's Rank correlation was used to evaluate correlations in this small cohort.

## 3 RESULTS

**Figure 1:**
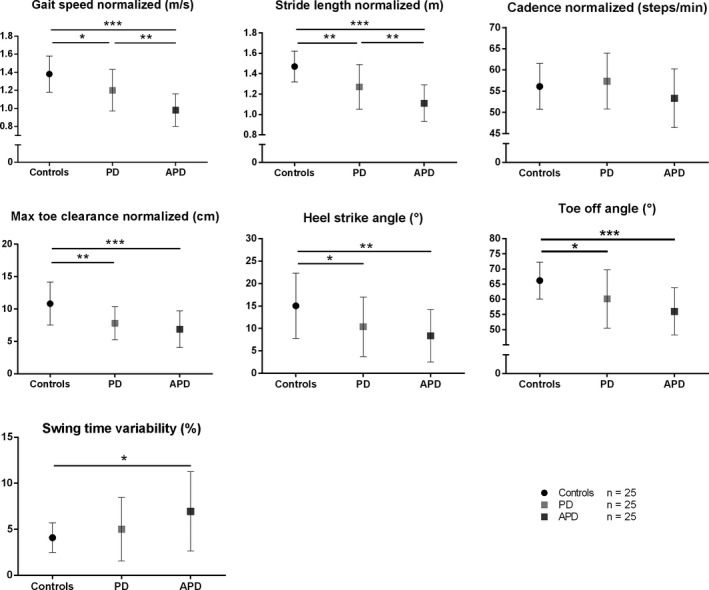
Spatiotemporal gait parameters (Mean ± *SD*) in patients with atypical Parkinson disorders (APD), patients with Parkinson's disease (iPD)—(matched by age, gender, age of onset, and Hoehn & Yahr disease stage), and healthy controls (matched by age and gender). Max toe clearance (cm), Maximum toe height during swing phase; Heel strike angle (°), Angle of heel contacting the floor at initiation of stance phase; Toe off angle (°), Angle of toe during push‐off at end of stance phase. * *p* < 0.05, ** *p* < 0.01, *** *p* < 0.001 Bonferroni post‐hoc test

**Figure 2:**
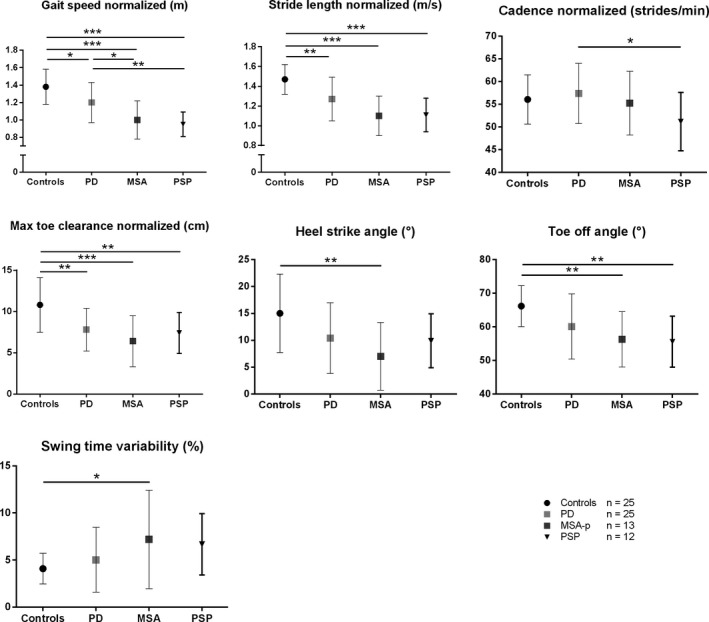
Spatiotemporal gait parameters (Mean ± *SD*) in patients with MSA, PSP, patients with idiopathic Parkinson's disease (iPD)—(matched by age, gender, age of onset and Hoehn & Yahr disease stage), and healthy controls (matched by age and gender). * *p* < 0.05, ** *p* < 0.01, *** *p* < 0.001 Bonferroni post‐hoc test

Patient characteristics are shown in Table 1. A detailed description of the gait parameters in each group is shown in Figure 1 and an overview of calculated gait parameters is shown in the supplementary Data S1. Gait speed *F*(2,72) = 23.955; * p*= .000) was significantly reduced in iPD patients (1.20 ± 0.23 m/s) compared to controls (1.38 ± 0.20 m/s; *p *=* *.011) and even more impaired in APD patients (0.98 ± 0.18 m/s; vs. controls *p *=* *.000 and vs. iPD *p *=* *.001). Similar results were obtained for stride length (*F*(2,72)=24.602; *p* = .000, controls 1.47 ± 0.15 m, iPD 1.27 ± 0.22 m and APD 1.11 ± 0.18 m; controls vs. iPD: *p *=* *.001, controls vs. APD: *p *=* *.000, iPD vs. APD: *p *=* *.007). Step cadence showed no significant difference between the groups. However, the maximum toe clearance (*F*(2,72)=12.486; *p* = .000) was significantly reduced in iPD (7.8 ± 2.6 cm; *p *=* *.001) and APD patients (6.9 ± 2.8 cm; *p *=* *.000) compared to controls (10.8 ± 3.3 cm) but did not reveal a significant difference between both patient cohorts. APD patients demonstrated a severely impaired heel strike angle (8.6 ± 5.8° *F*(2,72)=6.722; *p* = .002) compared to controls (15.0 ± 7.3°; *p *=* *.002) which was also present in iPD patients (10.4 ± 6.6°) vs. controls (*p *=* *.045), however, without significant difference between both groups. For toe off angles (*F*(2,72)=10.303; *p* = .000), we observed a significant reduction in iPD patients (60.1 ± 9.7°) compared to controls (66.2 ± 6.1°; *p *=* *.027), which was even more pronounced in APD patients (56.0 ± 7.8°, *p *<* *.001 vs. controls) without reaching significance compared to iPD patients (*p *=* *.213). Additionally, swing time variability (*F*(2,72)=4.838; *p* = .011) was significantly increased in APD (7.0 ± 4.3%) compared to controls (4.1 ± 1.6%; *p *=* *.010) but did not significantly differ compared to iPD patients (5.0 ± 3.4%; *p *>* *.05). We also analyzed MSA‐P and PSP patients separately, comparing them to iPD and controls. A detailed description is shown in Figure 2. Of note, gait parameters in MSA‐P and PSP did not differ significantly. Gait speed (*F*(2,72)=15.977: *p* = .000) was more reduced in MSA‐P (*p *=* *.035) and PSP (*p *=* *.005) compared to iPD patients, cadence (*F*(2,72)=2.710; *p* = .051) was even more reduced in PSP in comparison to iPD (*p *=* *.039), while heel strike angle (*F*(2,72)=4.902; *p* = .004) was more reduced in MSA‐P, compared to controls (*p *=* *.004) but not significantly reduced compared to iPD (*p *>* *.05).

**Table 1:** Patient characteristics

	PD	APD	Controls	*p*	APD subgroups	
					MSA	PSP
*n*	25	25	25	—	13	12
Age (y)	66.6 ± 7.9	65.4 ± 8.7	63.7 ± 9.7	>.05*	63.5 ± 8.5	67.4 ± 8.7
Gender (male:female)	13:12	13:12	13:12	—	4:9	9:3
Age onset (y)	58.1 ± 8.1	61.3 ± 7.5	—	>.05	60.3 ± 7.0	62.4 ± 8.2
Disease duration (y)	7.5 ± 4.5	4.0 ± 3.7	—	**<.01**	3.2 ± 3.9	5.0 ± 3.6
H&Y	2.7 ± 0.8	3.0 ± 0.4	—	>.05	3.0 ± 0.4	3.1 ± 0.3
MDS‐UPDRS‐3	31.7 ± 9.3	45.2 ± 12.8	—	**<.001**	48.6 ± 10.0	41.7 ± 15.5
LEDD (mg/d)	771.1 ± 509.0	671.8 ± 394.5	—	>.05	737.7 ± 504.1	600.4 ± 227.1

One‐way ANOVA (*followed by Bonferroni post‐hoc test), Significance level *p* < .05. Bold numbers indicate significance.

APD = Atypical Parkinsonian Disorders including; H&Y = Hoehn and Yahr disease stage; LEDD = Levodopa equivalent daily dose; MDS‐UPDRS‐3, (motor examination); MSA = Multiple system Atrophy; PD = Parkinson's disease; PSP = Progressive Supranuclear Palsy.

**Figure 3:**
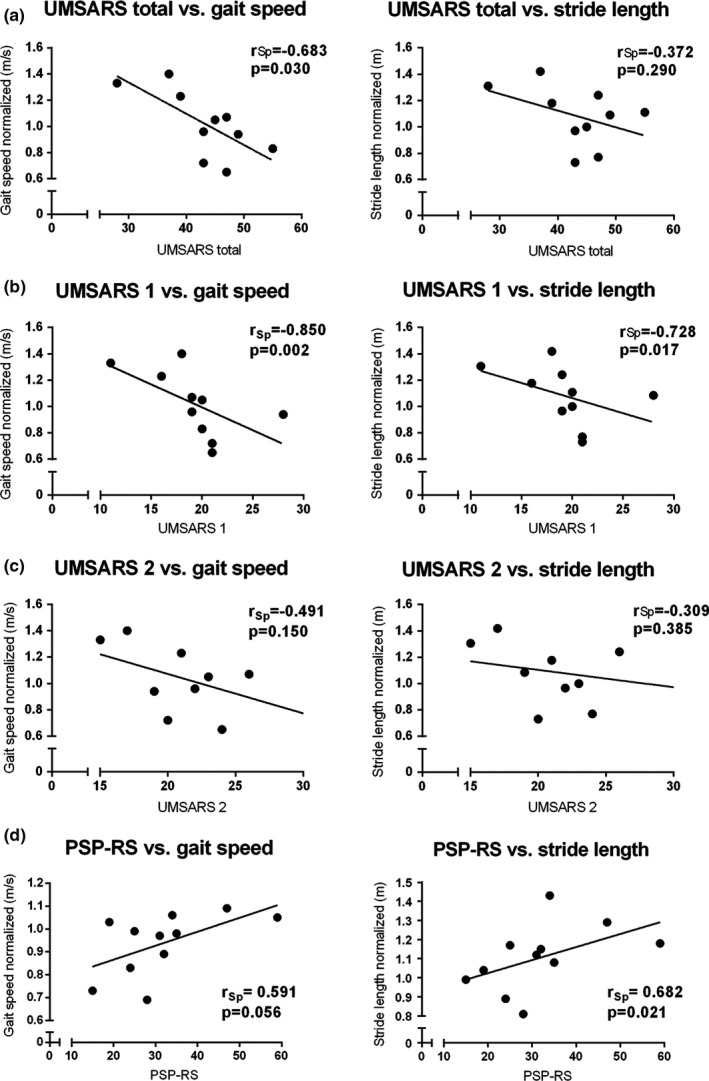
Correlations between spatiotemporal gait parameters (gait speed, stride length) and UMSARS total score, UMSARS‐1 (Historical review of motor and non‐motor symptoms) (a) as well as UMSARS‐2 (Motor examination) (b) in MSA patients. (c). Correlations between gait parameters (gait speed, stride length), and PSP‐RS in PSP patients (d). r_Sp_ = Spearman's rank correlation coefficient

**Figure 4:**
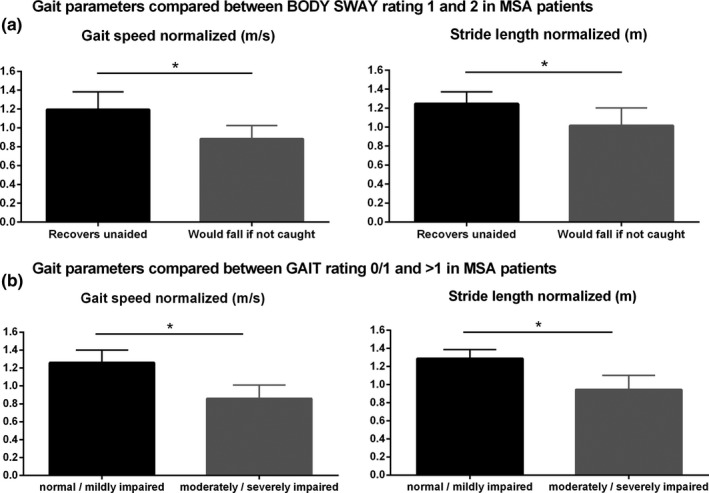
Comparison of gait parameters between (a) MSA patients with and without postural instability (BODY SWAY) and (b) between gait impairment levels rated by item gait of the UMSARS‐2. No/mildly (0/1) impaired gait and moderately/severely (>1) impaired gait in MSA patients were compared to objective gait parameters

According to our second goal, we correlated gait speed and stride length of patients with MSA‐P with the UMSARS total score, with the part 1 (=historical review of motor and non‐motor symptoms including walking, falling, and orthostatic symptoms; UMSARS‐1) and 2 (=Motor Examination without rating non‐motor symptoms, UMSARS‐2) (Figure 3a–c). Here, we observed a significant inverse correlation of gait speed and stride length with UMSARS total and UMSARS‐1 but not with UMSARS‐2 scores. According to the item 13 of the UMSARS‐2 (body sway), we divided MSA patients into two subgroups, namely patients who recovered unaided (e.g., 0–1 rating points) and patients who would fall if not caught (e.g., 2–4 rating points) and we compared these subgroups in terms of gait speed (*p* = .013) and stride length (*p* = .040), observing a statistically relevant difference (Figure 4a). Similarly, we divided ratings for the item gait (14 of UMSARS‐2) into normal/mildly impaired (e.g., 0–1 rating points) and moderately/severely impaired (e.g., 2–4 rating points). Here, a significant difference of the subgroups with gait speed (*p* = .011) and stride length (*p* = .011) was also shown (Figure 4b).

Moreover, stride length correlated with PSP‐RS scores in the PSP patients (*p* = .021) (Figure 3d). Finally, we observed a significant correlation of maximum toe clearance with MDS‐UPDRS‐3 (*r *=* *−.444, *p *=* *.026) in APD. In contrast, maximum toe clearance did not correlate with MDS‐UPDRS‐3 in iPD patients, whereas gait speed and stride length did.

## 4 DISCUSSION

Our study is the first that uses sensor‐based technology in APD patients, comparing objective spatiotemporal gait parameters with iPD and controls. All gait items except cadence showed gait and motor impairment in both parkinsonian cohorts compared to controls, similarly to a previous study in iPD patients (Schlachetzki et al., 2017). Among the different gait parameters, gait speed not only differentiated between controls and patients, but it was also more strongly reduced in APD compared to PD patients, despite similar global motor disability according to H&Y scores, indicating a more severe alteration of locomotor abnormality in APD patients. A similar tendency was shown for stride length. Our data demonstrate that these two gait parameters are a quantitative, metric measure for gait impairment and that they differ between APD and iPD. However, we were not able to discriminate MSA and PSP patients based on spatiotemporal gait parameters. This may reflect insufficient sample size, but also overlapping gait pathophysiology. Both MSA and PSP are characterized by levodopa refractory parkinsonism, impaired cerebellar outflow, and frontal lobe dysfunction all of which may contribute to gait disorders in APD patients (Wenning et al., 1999).

The second hypothesis of our study was that gait parameters correlate with clinical rating scores. Here, we observed a moderate correlation between MDS‐UPDRS‐3 and maximum toe clearance in APD patients. In contrast, we showed that gait speed and stride length correlated significantly with MDS‐UPDRS‐3 in iPD but not in APD. This finding may reflect the contribution of non‐parkinsonian impairments to the gait disorder of APD patients such as ataxia, orthostatic dysregulation, and frontal lobe impairment.

Moreover, we correlated gait speed and stride length of 10 MSA patients to the UMSARS total and specific clinical scores UMSARS‐1 and UMSARS‐2, respectively. We demonstrated a moderate inverse correlation with the UMSARS total score and a strong inverse correlation with the UMSARS‐1 but not with the UMSARS‐2. The UMSARS‐1 includes the historical review of motor and non‐motor symptoms including walking, falling, and orthostatic symptoms, which are main features of MSA and, in addition, markedly influence gait. On the contrary, the UMSARS‐2 includes the motor examination without considering non‐motor symptoms. We hypothesized that only UMSARS‐2 items “gait” and “body sway” are likely directly mirrored by objective gait parameters (Schlachetzki et al., 2017). Indeed, we observed a significant difference of gait speed and stride length between mildly and severely impaired MSA patients divided according to the single items “body sway” (UMSARS‐2 item 13) and “gait” (UMSARS‐2 item 14). However, these preliminary results in this small MSA cohort should be carefully interpreted and need further investigation.

Intriguingly, PSP in contrast to MSA and iPD patients revealed a positive correlation of disease severity as determined by PSP‐RS with gait speed and stride length. However, it should be noticed that three PSP patients used a gait support device, two of them a wheeled walker, the other one crutches. It has been shown that a four‐wheeled walker improves gait in iPD patients (Kegelmeyer et al., 2013) and in geriatric patients (Schülein et al., 2017). In our study, these two patients that used a wheeled walker showed the largest strides and highest gait speed within the PSP cohort indicating that the correlation is biased by the wheeled walker gait patterns.

We acknowledge that our APD patients' cohorts were rather small because of the rarity of these disorders. However, the significant difference of objective gait parameters among patient groups suggests that sensor‐based technology may support and complement the clinical assessment provided by validated rating scales. Longitudinal follow‐up studies in larger cohorts are needed to establish sensor‐based technology as outcome in trials and homecare.

## DISCLOSURE

This was not an industry‐supported study. This work was performed at the Department of Neurology, Innsbruck Medical University, Innsbruck, Austria and at the Department of Molecular Neurology, University Hospital Erlangen, Erlangen, Germany.

## CONFLICT OF INTEREST

Cecilia Raccagni declares a research grant from the MSA Coalition and from the Austrian Parkinson′s disease. Heiko Gaßner, Bjoern Eskofier, Jochen Klucken, and Juergen Winkler received institutional research grants from the Emerging Field Initiative of the Friedrich Alexander‐University Erlangen‐Nürnberg (EFI Moves, 2 Med 03), from the Bavarian State Ministry for Education, Science and the Arts, Munich, Germany (MotionLab@Home, E|Home Center), from the Bavarian Ministry of Economic Affairs and Media, Energy and Technology, Germany (Medical Valley Award 2016, Risk‐e‐Gait), and from the European Institute of Innovation and Technology (EIT Health, “MoveIT”). Sabine Eschlböck declares no conflict of interest. Sylvia Bösch reports grants from European Friedreich Ataxia Consortium for Translational Studies (EFACTS), FP7 Health (HEALTH‐F2‐2010‐242193), fees from serving on scientific advisory boards for Grünenthal, and Abbvie GmbH as well as honoraria from Ipsen, Allergan, Abbvie, Novartis, and Licher. Florian Krismer has received a research grant from the MSA Coalition, travel grants from the Austrian Parkinson's disease society as well as the International Parkinson's disease and movement disorders Society and non‐financial support from Fight MSA and Astra‐Zeneca, outside the submitted work. Klaus Seppi reports grants from Medical University Innsbruck, grants from Oesterreichische Nationalbank Nationalbank (Austrian Central Bank, Anniversary Fund; project no.: 14174), grants from Austrian Science Fund (FWF: Der Wissenschaftsfonds; project no.: KLI82‐B00), grants from the Michael J. Fox Foundation (project: PPMI study), personal fees from International Parkinson and the Movement Disorder Society, personal fees from Teva, personal fees from UCB, personal fees from Lundbeck, personal fees from AOP Orphan, personal fees from Roche and personal fees from Abbvie outside the submitted work. Werner Poewe reports personal fees from AbbVie, AstraZeneca, BIAL, Biogen, Britannia, Grünenthal, Intec, Ipsen, Lundbeck, Novartis, Neuroderm, Orion Pharma, Prexton, Sunovion, Teva, UCB and Zambon. (consultancy and lecture fees in relation to clinical drug development programmes for PD) outside the submitted work. Bjoern Eskofier holds ownerships of Portabiles HealthCare Technologies GmbH and Portabiles GmbH, received compensation and honoraria from serving on scientific advisory boards for Abbvie GmbH, Adidas GmbH, Bosch Sensortec GmbH, and ST Sportservice GmbH. Further, he gratefully acknowledges the support of the German Research Foundation (DFG) within the framework of the Heisenberg professorship programme (grant number ES 434/8‐1). Jürgen Winkler reports personal fees outside of the submitted work from Teva GmbH, Ever Pharma GmbH, Desitin Arzneimittel GmbH, Abbvie GmbH & Co. KG, Biogen GmbH, and GlaxoSmithKline GmbH & Co. KG. Gregor Wenning reports receiving consulting and/or lecture fees from Affiris, Astra Zeneca, Boehringer Ingelheim, Ever Pharma, Lundbeck, Neuropore, Orion and UCB as well as grant support from Medical University Innsbruck, Oesterreichische Nationalbank, FWF Austrian Science Fund, US MSA Coalition, Affiris, Astra Zeneca and Boehringer Ingelheim. Jochen Klucken holds ownerships of Portabiles HealthCare Technologies GmbH and Portabiles GmbH, received compensation and honoraria from serving on scientific advisory boards for LicherMT GmbH, Abbvie GmbH, UCB Pharma GmbH, GlaxoSmithKline GmbH & Co. KG, Athenion GmbH, and Thomashilfen GmbH; as well as lecturing from UCB Pharma GmbH, TEVA Pharma GmbH, Licher MT GmbH, Desitin GmbH, Abbvie GmbH, Solvay Pharmaceuticals, and Ever Neuro Pharma GmbH.

## AUTHORS CONTRIBUTIONS

Cecilia Raccagni: Research project: A. Conception, B. Organization, C. Execution; Manuscript: A. Writing of the first draft, B. Review and Critique. Heiko Gaßner: Statistical Analysis: A. Design, B. Execution, C. Review and Critique; Manuscript: A. Writing of the first draft, B. Review and Critique. Sabine Eschlboeck: Research project: C. Execution. Sylvia Boesch: Manuscript: C. Review and Critique. Florian Krismer: Manuscript: C. Review and Critique. Klaus Seppi: Manuscript: C. Review and Critique. Werner Poewe: Manuscript: C. Review and Critique. Bjoern M. Eskofier: Manuscript: C. Review and Critique. Jürgen Winkler: Statistical Analysis: C. Review and Critique; Manuscript: C. Review and Critique. Gregor Wenning: Research project: A. Conception; Manuscript: C. Review and Critique. Jochen Klucken: Research project: A. Conception, C. Execution; Manuscript: C. Review and Critique.
