# Supplementary material for: Sensor‐based gait analysis in atypical parkinsonian disorders
Source: Brain Behav. 2018 May 7;8(6):e00977. doi: 10.1002/brb3.977 (PMC5991583; doi:10.1002/brb3.977)
Supplement: Supplementary file 1 [file BRB3-8-e00977-s001.docx]

| **Gait Parameter** | **Definition** |
| --- | --- |
| Stride length [m] | Distance between two sequential points of mid stance contacts with the same foot |
| Stride time [s] | Duration of one gait cycle |
| Gait velocity [m/s] | Walking speed in a designated direction |
| Cadence [spm] | Stride rate per minute |
| Stance phase time [%] | Period in gait when the foot is in contact with the floor |
| Swing phase time [%] | Period in gait cycle when the foot is *not* in contact with the floor |
| Max. toe clearance [cm] | Maximum toe height during swing phase |
| Heel-strike angle [°] | Angle between foot and ground in the sagittal plane at heel-strike |
| Toe-off angle [°] | Angle between foot and ground in the sagittal plane at toe-off |
| Gait variability [%] | Coefficient of variance (SD / Mean * 100) |

**Supplementary material**

**Table S1.** Definition of gait parameters

Abbreviations: m=meter; m/s=meter per second; spm=strides per minute; cm=centimeter;

**
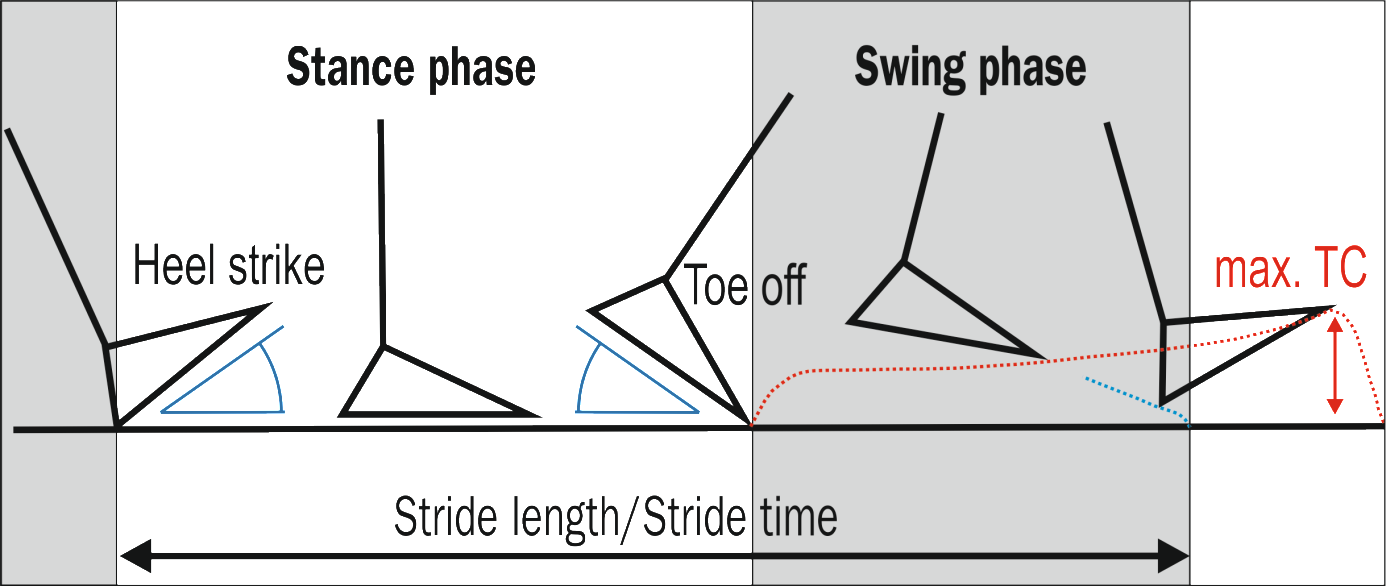
**

**Figure S1: Overview of calculated gait parameters.** Spatiotemporal gait parameters were extracted from gait patterns using signal processing algorithms. Characteristic gait parameters of the gait cycle are stride length/stride time, stance and swing phase, heel strike and toe off angles as well as maximum toe clearance (max. TC). Additionally gait velocity and cadence (strides per minute) were calculated based on gait signals.
